# Safety of percutaneous dilatational tracheotomy (PDT) with the rigid tracheotomy endoscope (TED): a 6-month follow-up multicenter investigation

**DOI:** 10.1186/s12871-021-01264-2

**Published:** 2021-02-15

**Authors:** Andreas Nowak, Eckart Klemm, Caroline Michaelsen, Taras I. Usichenko, Sven Koscielny

**Affiliations:** 1 Head of the Department of Anesthesiolgy & Intensive Care Medicine, Emergency Medicine & Pain Management, Dresden Municipal Hospital - Academic Teaching Hospital of the Dresden University of Technology, Friedrichstrasse 41, 01067 Dresden, Germany; 2 Department of Otorhinolaryngology, Head and Neck Surgery, Plastic Surgery, Dresden Municipal Hospital - Academic Teaching Hospital of the Dresden University of Technology, Dresden, Germany; 3 Department of Anesthesiology, Intensive Care Medicine, Emergency Medicine, Pain Medicine, University Medicine of Greifswald, Greifswald, Germany; 4 Department of Anesthesia, McMaster University, Hamilton, Canada; 5 Department of Otolaryngology and Institute of Phoniatry and Pedaudiology, Jena University Hospital, Jena, Germany

**Keywords:** Tracheotomy, Percutaneous dilatational tracheotomy, Rigid endoscopy, Late complications, Tracheal stenosis

## Abstract

**Background:**

The rigid tracheotomy endoscope (TED) was recently introduced to improve the fiberoptic technique during percutaneous dilatational tracheotomy (PDT) in critically ill patients. The aim was to evaluate the long-term complications of PDT using TED equipment in a prospective multicenter investigation.

**Methods:**

One hundred eighty adult patients underwent PDT using TED in four German hospitals. Patients who were alive or their guardians were contacted via telephone and interviewed using a structured questionnaire 6 months following the tracheostomy procedure. Patients with airway complaints were invited for outpatient clinical ENT examination. The incidence of adverse events related to PDT was registered.

**Results:**

Of 180 patients who received tracheostomy, 137 (76.1%) were alive at the time of follow-up. None of the 43 lethal events was related to the PDT. Fifty-three (38.7%) patients were available for follow-up examination, whereas 14 (10.2%) were able to visit ENT physicians. Two (3.8%) out of 53 patients developed tracheocutaneous fistula with required surgical closure of tracheostoma. Dyspnea (7.5%), hoarseness (5.7%), stridor and swallowing difficulties (both with 3.8%) were the most common complaints. Tracheal stenosis was confirmed in 1 patient (1.88% [95% CI: 0.33; 9.93]).

**Conclusion:**

The use of TED for PDT in the clinical setting is safe regarding adverse events at 6-month follow-up. The incidence of tracheal stenosis after PDT with TED is comparable with that of flexible bronchoscopy; however, its role for PDT at the intensive care unit should be clarified in further investigations.

**Supplementary Information:**

The online version contains supplementary material available at 10.1186/s12871-021-01264-2.

## Background

Percutaneous dilatational tracheotomy (PDT), performed in the ICU, is considered the procedure of choice to establish the tracheostomy airway in critically ill adult patients [1]. As an alternative to open surgical tracheotomy (OST), PDT has been increasingly used for temporary access to the trachea in the intensive care unit because it is associated with a low complication rate and is at least as safe as surgical tracheotomy in the ICU setting [2, 3]. Patients with an expected short ventilation period in the ICU are likely to benefit from PDT since it can be performed with less effort than OST directly at the bed side in the ICU. The stoma usually closes spontaneously after removal of the tracheostomy cannula within a short time without additional intervention; the long-term aesthetic results are superior to those of OST [2].

A recent retrospective observational study in nursing homes revealed that in 66% of patients, PDT was used to establish the airway. The late complication rates of PDT were significantly higher for all complication types than for patients receiving OST care. Eighty percent of patients with PDT vs. 23% with OST required readmission to a hospital for tracheostoma revision [3]. Recent systematic review demonstrated that PDT can be associated with severe early and late complications, such as hemorrhage, loss of airway, injury to posterior tracheal wall and via falsa including death [4].

Fiberoptic tracheo-bronchoscopy remains the standard method to perform PDT [1, 2]. Obstruction of the endotracheal tube due to flexible endoscope during PDT may cause ventilation problems with subsequent hypoxemia, hypercarbia, increased intracranial pressure and pneumothorax [5]. In order to provide better visualization of the tracheal anatomy and improve airway management and safety during the PDT procedure, the rigid tracheotomy endoscope (TED) was introduced [6]. The use of TED-based PDT in 180 patients was comparable to that using flexible bronchoscopy in terms of safety issues, offering the opportunity for additional jet ventilation during PDT [7], which has advantages in preventing blood aspiration in case of intratracheal bleeding [8]. However, the potential late complications of PDT, such as tracheal stenosis, remained beyond the scope of this prospective multicenter investigation [7].

Thus, the aim of this present investigation was to evaluate the long-term potential complications of PDT using TED equipment 6 months following tracheostomy procedure.

## Materials and methods

### Study design and patient selection

This study was a follow-up observational investigation recruiting all patients from the prospective multicenter investigation of the safety and feasibility of PDT with TED [7]. Briefly, after approval of the local ethics commission, 180 adult patients in intensive care and those scheduled for ENT surgery underwent PDT using TED in four German hospitals: Hospital Dresden-Friedrichstadt (city of Dresden), Cardiovascular Center (city of Cottbus), Hospital Ernst von Bergmann (city of Potsdam) and Hospital Chemnitz (city of Chemnitz). PDTs were performed in mixed teams of intensivists, surgeons and ENT physicians. Detailed characteristics of these teams are given in Supplementary Table 1. The exclusion criteria were age < 18 years, emergency cases, primary critical oxygenation parameters, severe gastroesophageal reflux disease, anatomical peculiarities (large thyroid goiter, fixed cervical spine, herniated discs and instability of the cervical spine), difficult airway, coagulopathy with an international normalized ratio (INR) < 1.5 and platelet count ≤50 Gpt/l and phlegmonous inflammation of the neck. All patients (for unconscious patients, the legal guardian) gave their written informed consent to participate, including the follow-up investigation, which was performed 6 months after the PDT procedure.^7^

### Data collection and analysis

The ENT physician (CM) contacted the patients (or their guardians) via telephone and interviewed them using the structured questionnaire (Additional file 1). This questionnaire contained the following items: 1) if the patient was deceased (with any association with PDT); 2) if the tracheal incision was closed; 3) if tracheal incision required subsequent neck surgery or any other therapy regarding PDT problems was necessary; and 4) if the following symptoms occurred after PDT: i) dyspnea; ii) stridor; iii) dysphagia; iv) hoarseness; v) bleeding from tracheostoma; vi) local inflammation; and vii) difficulties with tracheostomy tube exchange.

Patients (or their guardians) who reported the pathologic symptoms specific for tracheal stenosis during the interview and who were able to be transported were invited for outpatient clinical examination. This examination was performed by an ENT physician and included flexible translaryngeal tracheoscopy to clarify the origin of the symptoms. The descriptive data were managed using IBM SPSS Statistics Software for Mac (Version 19.0.0, IBM Corp., New York, USA) and are presented as the mean (standard deviation) and number (percent) unless otherwise stated.

## Results

### Patients available for follow-up

**Fig. 1:**
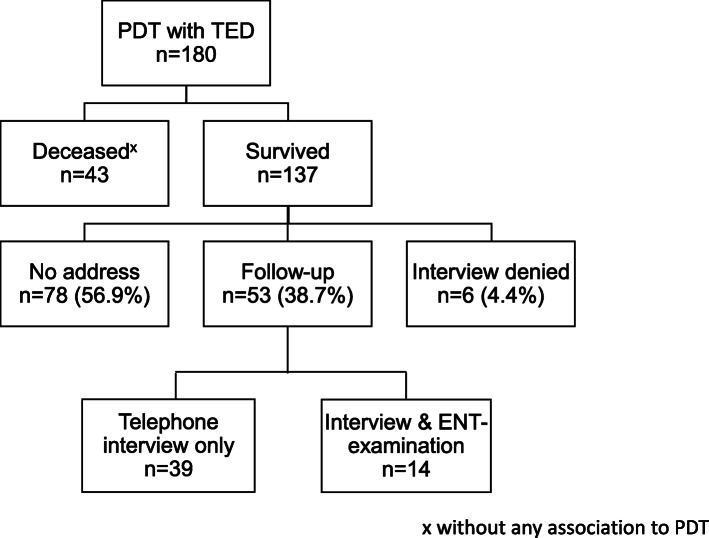
Enrollment flowchart

Of 180 patients who initially received tracheostomy, 137 (76.1%) were alive at the time of follow-up (Fig. 1). Out of 43 deceased patients, 27 died in the hospital, and 16 died after discharge within 6 months following PDT. None of these lethal events were related to PDT (Table 1). Fifty-three out of 137 (38.7%) patients were available for follow-up examination, whereas 14 (10.2%) were able to visit ENT physicians, where fiberoptic translaryngeal tracheoscopy was carried out (Fig. 1). In 2 patients (3.8%), retracheotomy was necessary after tracheostoma closure. The indications for tracheotomy resulted from pneumonia and edema due to radiotherapy. Detailed demographic and clinical characteristics of these 53 patients available for follow-up examination are given in Supplementary Table 2.

**Table 1:** Causes of death during follow-up period (*n* = 43)

Causes of death	***n*** (%)
Pneumonia	13 (30.2)
Multiorgan failure	12 (27.9)
Sepsis	12 (27.9)
Underlying cancer disease	2 (4.7)
Ischemic stroke	1 (2.3)
Ischemic colitis	1 (2.3)
Cardiogenic shock	1 (2.3)
Bleeding due to recurrent bronchial cancer	1 (2.3)

### Late complications of PDT

Two (3.8%) out of 53 patients developed tracheocutaneous fistula with required surgical closure of the tracheostoma. In 1 case, decannulation was not possible due to supraglottic edema after radiotherapy (Table 2). There were no serious complications of PDT requiring additional treatment, such as local inflammation, difficult exchange of tracheostomy tubes and bleeding. Dyspnea in 4 patients (7.5%), hoarseness in 3 (5.7%), and stridor and swallowing difficulties in 2 patients (both with 3.8%) were the most common complaints among 53 patients from the follow-up collective.

**Table 2:** Items of questionnaire at 6 months PDT follow-up (*n* = 53)

Items	***n*** (%)
Death related with PDT	0
Closure of tracheostoma	
spontaneous	50 (94.3)
surgical	2 (3.8)
Decannulation not possible (supraglottic edema)	1 (1.9)
Symptoms	
dyspnea	4 (7.5)
hoarseness	3 (5.7)
stridor	2 (3.8)
swallowing disorder	2 (3.8)
bleeding from tracheostoma	0
difficult exchange of tracheostomy tube	0
local inflammation	0

### Tracheal stenosis after PDT

In all 4 patients (all males) who reported dyspnea on exertion, PDT was performed between the 2nd and 3rd tracheal rings using the Ciaglia Blue Rhino technique, and the tracheostoma was spontaneously closed (Table 3). Two of these patients also reported dyspnea at rest as well as stridor during breathing. One of the patients (Patient 1, Table 3) had inflamed tracheal mucosa prior to PDT. The other patient (Patient 3) who complained of dyspnea at rest and stridor during breathing developed supraglottic edema due to radiotherapy, which was given to prevent the malignant growth of underlying laryngeal cancer (Table 3). In this patient, no tracheal stenosis was confirmed using flexible translaryngeal tracheoscopy. In the first patient (Patient 1) with stenotic complaints, a tracheal ring fracture occurred during PDT. Tracheal stenosis was confirmed in this patient using flexible translaryngeal tracheoscopy (Patient 1). Thus, the frequency of functionally relevant tracheal stenosis after PDT with TED was 1.88% (95% CI: 0.33; 9.93).

**Table 3:** Clinical features of four patients with dyspnea 6 months after percutaneous dilatational tracheotomy (PDT)

Feature	Patient 1	Patient 2	Patient 3	Patient 4
Underlying disease	Ileus	Colon cancer	Laryngeal cancer	COPD
Concomitant condition	–	–	Supraglottic edema due to radiotherapy	–
Duration of endotracheal intubation prior to PDT (days)	9	14	0	8
Indication for PDT	1	1	2	1
Trachea inflammation prior to PDT	+	+	–	–
Tracheal ring fracture due to PDT with subsequent resection	+	–	–	–
Dyspnea				
at rest	+	–	+	–
on exertion	+	+	+	+
Stridor				
inspiration	+	–	+	–
expiration	+	–	+	–

*COPD* Chronic obstructive pulmonary disease; 1: prolonged ventilation; 2: securing the airway for subsequent ENT surgery

## Discussion

The use of rigid endoscopy in different technical variants for performing PDT was reported as feasible and safe regarding possible early side effects and complications [6, 9–13]. Long-term complications following PDT often remain beyond the sight of the intensive care physician. To date, there are no studies evaluating late complications after PDT with rigid endoscopy. The present prospective investigation is the first endeavor to summarize such complications.

No local inflammation, difficult exchange of tracheostomy tubes or bleeding requiring additional treatment were observed in our investigation. Breathing symptoms such as dyspnea (7.5% of cases), hoarseness (5.7%) and stridor (3.8%) were the most common complaints among 53 patients from the follow-up cohort. A retrospective study 6 years following PDT reported the incidence of severe hoarseness in 11% and severe breathing difficulties in 3.3% of patients [14]. These clinical symptoms may be indicators of tracheal stenosis. Tracheal stenosis is likely to be symptomatic only in severe cases, where lumen constriction from 60 to 70% up to total occlusion (grade III and IV according to Myer and Cotton) is present [15]. A nationwide investigation in the USA revealed an incidence of 1.05% for tracheal stenosis due to tracheostomy [16]. Our data are comparable to previous studies that showed an incidence of tracheal stenosis after PDT of 1–6% [17–24].

Clear differentiation of causality in the development of tracheal stenosis is not always possible. The causes of tracheal stenoses are complex and usually represent a combination of tracheal trauma, inflammation and foreign body irritation with tissue formation (granulation) at predisposed sites above, next to and below the stoma with loss of the original tracheal tissue layer by fibrosis [25]. The ring cartilage reacts particularly sensitively to local trauma with the development of recurrent tracheal stenosis caused by excessive regeneration processes with osteoid expression of osteoblasts and mineralization in an acidic environment [25]. Beyond the tracheotomy technique, overweight, diabetes and reflux, accompanied by chronic inflammatory reactions, are risk factors for the development of subglottic stenosis [26]. A recent analysis of 262 cases suggested that COPD, nicotine abuse, OSAS, hypertension and microcirculation disorders are the comorbidities responsible for the development of laryngotracheal stenosis following tracheostomy [27]. The incidence of tracheal stenosis after tracheostomy and endotracheal intubation is significantly higher in keloid than in nonkeloid subjects [28]. In our investigation, we found two patients with dyspnea at rest as well as stridor during breathing. One of them developed inflammation of the tracheal mucosa prior to PDT, and a tracheal ring fracture occurred during PDT. Tracheal ring fracture represents a significant local trauma. There is no conclusive opinion on the causality between tracheal ring fractures and tracheal stenoses. It does not escape our notice that the tracheal braces do not have homogeneous histomorphic structures, as the examinations on 103 tracheotomized patients in intensive care medicine showed. There are numerous histological formations that may facilitate brace fractures during PDT. In 25% of tracheal braces, advanced ossification was observed in the central parts, which eliminated the elasticity of the tracheal braces. This negatively influences the mechanical stability of the trachea and the elasticity of the cartilage braces and seems to be a disposition for fractures of braces in PDT [29].

After percutaneous dilatational tracheotomy, the stoma usually closes spontaneously within three to 5 days after decannulation in almost 100% of cases [2]. A period of months before decannulation can result in epithelialization of the tracheostoma and later in the formation of a tracheocutaneous fistula [30]. In two patients with tracheocutaneous fistulas, the times from PDT to decannulation were 179 and 274 days, respectively. Our results regarding a persistent tracheocutaneous fistula are comparable to those reported in the literature [30, 31].

It is known that follow-up investigations in former ICU patients are difficult for various reasons [30]. In our study, almost 24% of patients died during the follow-up period, and more than half of the surviving patients could not be reached for the follow-up questionnaire, whereas only 4.4% of them refused the telephone interview. Thus, our data on the frequency of response of patients to follow-up examination after PDT are in agreement with the literature on that topic, which gives response rates from 23% [30] to a maximum of 60% (Table 4).

**Table 4:** Data about tracheal stenoses after PDT from follow-up investigations with telephone interview and questionnaire

Authors (year)/reference	Design	Number of patients	Tracheotomy method	Number of patients with tracheal stenosis, (%)	Time of follow-up (months)	Type of follow-up
Hill et al. (1996) [18]	prospective	p 353 f 214	PDT (C)	symptomatic stenosis 8 (3.7)	10	telephone interview, clinical examination
Law et al. (1997) [19]	prospective	p 109 f 41	PDT (C)	stenosis > 40% 1 (2.4)	6	telephone interview, spirometry, endoscopy
Rosenbower et al. (1998) [20]	prospective	p 95 f 55	PDT (C)	subglottic stenosis 2 (2.0)	12	endoscopy ENT, telephone interview
Norwood et al. (2000) [17]	prospective	p 422 f 100	PDT (C)	stenosis > 50% 3 (3.0)	26	telephone interview, endoscopy, CT
Escarment et al. (2000) [21]	prospective	p 162 f 81	GWDF (G)	surgery due to stenosis 4 (4.9)	3	clinical visit, endoscopy, telephone interview
Dollner et al. (2002) [22]	retrospective	p 60 f 19	GWDF (G)	stenosis > 25–50% 2 (3.3) stenosis > 50% 1 (1.6)	17	telephone interview, clinical examination, endoscopy
Young et al. (2014) [23]	prospective	p 120 f 50	PDT (B)	stenosis > 46% 5 (4.0)	3	questionnaire, MRI, spirometry

B: PDT acc. to Ciaglia Blue Rhino, p: number of patients who received PDT

C: PDT acc. to Ciaglia, f: number of patients, available for follow-up examination

G: GWDF acc. to Griggs, *CT* Computerized tomography

*MRI* Magnetic resonance imaging

### Limitations

The main limitation of our investigation is the scarce responses of surviving patients to follow-up interviews due to the long-term observational design of the study and lethality from the underlying disease. The inability to reach patients for an interview due to a change in their residence may lead to a false low incidence of late complications following PDT with TED. Moreover, our questionnaire was based on subjective symptoms surveyed in telephone interviews, which may have introduced bias into the results. There is no clear unified definition of tracheal stenosis, making the comparability of follow-up examinations difficult. Finally, for lung ventilation, the trachea was intubated in patients prior to PDT; hence, possible subsequent airway injury may serve as a confounding factor in reviewing the long-term adverse events following tracheostomy.

## Conclusions

Regarding the complications at 6-month follow-up, the use of TED for PDT in the clinical setting is safe. Functionally relevant tracheal stenoses following PDT are possible and may remain beyond the view of the intensivist. The incidence of tracheal stenoses after PDT with TED is comparable with that of flexible bronchoscopy. The differentiation between technical causes and pathogenetic factors in the development of tracheal stenoses after PDT is not possible in most cases. Prospective studies with larger sample sizes would be helpful to identify the risk factors for potential complications and to compare various PDT techniques with this purpose. The question whether the use of TED during PDT at the intensive care unit may reduce the rate of long-term complications should be addressed to randomized clinical trials.

## Supplementary Information

**Additional file 1: Supplementary Table 1**. Expertise of the PDT teams. **Supplementary Table 2**. Demographic and clinical characteristics of follow-up collective (*n* = 53). **Appendix 1**. Telephone questionnaire regarding late complications after PDT with TED

## Data Availability

The datasets generated during and analyzed during the current study are not publicly available due to due to relevant data protection laws but are available from the corresponding author on reasonable request.
