# Supplementary material for: Safety of percutaneous dilatational tracheotomy (PDT) with the rigid tracheotomy endoscope (TED): a 6-month follow-up multicenter investigation
Source: BMC Anesthesiol. 2021 Feb 15;21:51. doi: 10.1186/s12871-021-01264-2 (PMC7883418; doi:10.1186/s12871-021-01264-2)
Supplement: Supplementary file 1 — Additional file 1: Supplementary Table 1. Expertise of the PDT teams. Supplementary Table 2. Demographic and clinical characteristics of follow-up collective (n = 53). Appendix 1. Telephone questionnaire regarding late complications after PDT with TED [file 12871_2021_1264_MOESM1_ESM.docx]

**Safety of percutaneous dilatational tracheotomy (PDT) with the rigid tracheotomy endoscope (TED): a 6-month follow-up multicenter investigation**

**Names of authors:**

Andreas Nowak*, MD

Head of the Department of Anesthesiology & Intensive Care Medicine, Emergency Medicine & Pain Management

Municipal Hospital Dresden - University Teaching Hospital

Dresden, Germany

Eckart Klemm, MD,

Department of Otorhinolaryngology, Head and Neck Surgery, Plastic Surgery

Municipal Hospital Dresden - University Teaching Hospital

Dresden, Germany

Caroline Michaelsen, MD

Department of Otorhinolaryngology, Head and Neck Surgery, Plastic Surgery

Municipal Hospital Dresden - University Teaching Hospital

Dresden, Germany

Taras I. Usichenko, MD,

Department of Anesthesiology, Intensive Care Medicine, Emergency Medicine, Pain Medicine

University Medicine of Greifswald
Greifswald, Germany

Department of Anesthesia

McMaster University

Hamilton, Canada

Sven Koscielny, MD

Department of Otolaryngology and Institute of Phoniatry and Pedaudiology

Jena University Hospital

Jena, Germany

**Running title:** Follow up after dilatational tracheotomy with rigid endoscopy

***Correspondence to:** Andreas Nowak, MD

Head of the Department of Anesthesiology & Intensive Care Medicine, Emergency Medicine & Pain Management

Dresden Friedrichstadt Hospital

Friedrichstrasse 41, 01067 Dresden, Germany

E-mail: andreas.nowak@klinikum-dresden.de

Phone: +49 351 480 1170

Fax: +49 351 480 1179

Supplementary table 1. Expertise of the PDT teams

| **Endoscopy** | **Puncture** | **n (%)** |
| --- | --- | --- |
| ENT | INT | 99 (55.0) |
| ENT | ENT | 41 (22.7) |
| ENT | NS | 26 (14.5) |
| INT | ENT | 8 (4.5) |
| NS | ENT | 3 (1.7) |
| INT | INT | 2 (1.1) |
| ENT | S | 1 (0,5) |

INT: Intensivist ENT: ENT physician
NS: Neurosurgeon S: Surgeon

Supplementary table 2. Demographic and clinical characteristics of follow-up collective (n=53)

| **Characteristic** | **Value** |
| --- | --- |
| Age, years | 57.1 ± 15.4 |
| Gender, f/m | 14 (27.5) / 37 (72.5) |
| Body mass index (kg/m^2^) | 26.3 ± 5.0 |
| Underlying disease/cause of PDT |  |
| laryngeal cancer | 14 (26.4) |
| sepsis | 8 (15.1) |
| prolonged weaning following surgery | 7 (13.2) |
| pneumonia | 4 (7.5) |
| polytrauma | 3 (5.7) |
| intracranial bleeding | 2 (3.8) |
| esophageal cancer | 2 (3.8) |
| hemorrhagic shock | 2 (3.8) |
| cardiopulmonary resuscitation | 2 (3.8) |
| traumatic brain injury | 1 (1.9) |
| acute pancreatitis | 1 (1.9) |
| exacerbated COPD | 1 (1.9) |
| ileus | 1 (1.9) |
| mediastinitis | 1 (1.9) |
| Crohn disease | 1 (1.9) |
| thoracic trauma | 1 (1.9) |
| Comorbidities |  |
| arterial hypertension | 17 (32.1) |
| COPD | 6 (11.3) |
| metabolic disorder | 9 (17) |
| immunological disorder | 1 (1.9) |
| Type of PDT procedure |  |
| Blue Rhino | 52 (98.1) |
| GWDF | 1 (1.9) |
| Laryngeal pathology prior to PDT |  |
| edema | 3 (5.7) |
| inflammation | 1 (1.9) |
| Tracheal pathology prior to PDT |  |
| edema | 1 (1.9) |
| inflammation | 19 (35.8) |
| Tracheal ring fracture due to PDT | 5 (9.4) |

Values are given as mean ± standard deviation or number of patients (percent)

COPD: chronic obstructive pulmonary disease; GWDF: guide wired delated forceps tracheostomy

**Appendix 1**

**Telephone questionnaire regarding late complications after PDT with TED**

Telephone interview declined

*Identity assurance*

1. Name:

2. Date of birth:

3. Interview with:

*Current state of the patient*

1. Current condition:

2. Current problems:

3. Legal guardian:  yes  no

4. Tracheostoma closed:  yes  no

5. Patient died:  yes  no

6. If yes:

- When (month):

- What was the process after being discharged?:

- Has the patient died  at home  in the rehabilitation clinic  in the hospital?

- Cause of death:

- Tracheotomy-related death:

*Findings*

1. Any problems with the Tracheotomy?  yes  no

2. If yes, which ones?

3. Was treated?

4. By whom was treated (name of the ENT doctor or family doctor)?

*Special questions*

1. Shortness of breath a) at rest?  yes  no

b) on exertion?  yes  no

2. Wheezing breath a) when inhaling?  yes  no

b) when exhaling?  yes  no

3. Is bronchial asthma known?  yes  no

4. Operation on the neck necessary due to tracheotomy?  yes  no

5. Any problems with swallowing?  yes  no

- if yes, which:

6. Any problems with speaking?  yes  no  hoarseness

is loud speaking possible?  yes  no

7. Any bleeding from the trachea?  yes  no

- If so, when:

- treatment by whom:

8. Have you had problems changing the cannula?  yes  no

- if yes, which:

9. Inflammation at the tracheotomy?  yes  no

10. Retracheotomy necessary after closure?  yes  no

- if yes, why:

11. Is/was treatment for the tracheotomy by an ENT doctor required?  yes  no

- if yes, why:

12. Was re-treatment in the hospital necessary due to tracheotomy?  yes  no

- Name of the hospital:

15. Name of the otolaryngologist:

16. Name of the general practitioner:
